# An assessment of cryptocurrencies as a global commercial determinant of health

**DOI:** 10.1093/heapro/daae190

**Published:** 2024-12-17

**Authors:** Nathan Davies

**Affiliations:** Nottingham Centre for Public Health and Epidemiology, School of Medicine, University of Nottingham, Hucknall Rd, Nottingham NG5 1PB, UK

**Keywords:** commercial determinants of health, gambling, cryptocurrency, marketing, global, sportswashing, regulation

## Abstract

Through the commercial determinants of health framework, gambling has been identified as a powerful threat to health. This research critically examines cryptocurrency, which is promoted and sold as a highly gamblified product. Using the commercial determinants of health framework, the multifaceted ways in which cryptocurrency firm operations may impact health outcomes are highlighted. Political influence is exerted through substantial donations, with high-profile cases illustrating the sector’s attempts to sway policy, whilst cryptocurrencies often operate in unregulated markets. Marketing strategies mirror those of traditional harmful industries, deploying immense advertising budgets and celebrity endorsements to promote highly speculative and risky financial products. Cryptocurrency mining, demanding considerable energy consumption, causes significant environmental damage. Financial practices include hundreds of outright frauds targeting low- and middle-income countries. Cryptocurrency investment, with 24/7 access and promises of huge wealth, mirrors gambling and is likely to result in public health harms through the same mechanisms as other forms of gambling. Despite the supposed potential of blockchain technology for improving payment and contract systems, the lack of realization of these benefits contrasts sharply with the immediate and growing costs associated with cryptocurrency speculation. Cryptoassets are a case study for the need for health promotion professionals to critically evaluate new technologies and advocate for regulatory measures to protect public health in the face of novel, high-risk products that overlap gambling and finance.

**Contribution to Health Promotion**

- Demonstrates the link between gambling and cryptocurrencies.
- Describes the business practices of cryptocurrency firms, including large-scale sports advertising, hidden business structures and political donations.
- Highlights the health harms from these products, including huge financial losses to individuals, environmental damage and poorer mental health.
- Outlines the cases for further research and exploration of strong regulation for gamblified financial products that cause health harms.

## INTRODUCTION

It has been long established that the activities of commercial actors have a significant influence on health (Kickbusch *et al*., 2016). The Lancet’s recent three-part series on commercial determinants of health set out the case for focusing on a broader range of commercial and quasi-commercial entities that impact health, both in terms of risks and benefits (Lacy-Nichols *et al*., 2023). The authors define the commercial determinants of health as ‘the systems, practices, and pathways through which commercial actors drive health and equity’ (Gilmore *et al*., 2023). This model helps identify how under-regulated industry practices can harm health in hidden ways, and the systemic issues that allow these practices to continue.

The scope of commercial determinants of health is expanding beyond its traditional focus on tobacco, alcohol and unhealthy foods. One area of substantial recent interest to commercial determinants of health scholars has been gambling (McCarthy *et al*., 2020; Schalkwyk *et al*., 2021; Thomas *et al*., 2023). Gambling has been established as having a negative influence on a range of the wider determinants of health (Johnstone and Regan, 2020). It can damage the health of those who use its products and their families in a multitude of ways, including financial difficulties, family functioning, poorer mental health, and increased use of alcohol and other drugs (Browne *et al*., 2016; Public Health England, 2021; Marko *et al*., 2023). Products that could be described as gambling-alike, gambling-adjacent or gamblified, are high-priority for assessment through a commercial determinants of health framework. One group of high-profile products fitting this description are cryptoassets, such as cryptocurrency and non-fungible tokens (NFTs).

### Understanding cryptoassets and cryptocurrencies

Cryptoassets are described as ‘digital representations of value or rights’, secured by encryption and distributed ledger technology, which is a type of blockchain technology (Financial Conduct Authority, 2024). Blockchain technology allows every cryptocurrency or NFT transaction to be recorded and published across records, or ‘ledgers’, held by all network members. This enables cryptoassets to operate without the support of central banks or governments, providing a degree of anonymity for users and, arguably, a secure record of transactions (Feyen *et al*., 2022). There are thousands of cryptocurrencies and NFT providers. This includes the well-known Bitcoin, the first cryptocurrency, but also so-called stablecoins linked to other assets (like the dollar), and thousands of unstable speculative small-scale cryptocurrencies that surface regularly (CoinMarketCap, 2023). To some purchasers, the appeal of cryptocurrency is to have a form of payment that does not rely on a government or bank; to others, it is an investment they hope will make them money by increasing in value before they sell it on (Financial Conduct Authority, 2023b). Cryptoassets have avoided significant regulation in many jurisdictions by being classified as neither a financial product nor a gambling product (House of Commons Treasury Committee, 2023).

### Current understanding of health harm of cryptocurrencies

Financial product products that enable high-frequency trading often exhibit many of the features of gamblification. Most investors lose, they are attractive to those who also experience gambling-related harm, and products are either designed for high-frequency use or advertise huge returns (Newall and Weiss-Cohen, 2022; Andrade and Newall, 2023). A significant proportion of cryptocurrencies fall squarely into this category. Unlike regular trading, which is restricted by opening hours and transaction limits, cryptocurrency trading usually has 24-h, 365-day accessibility (Andrade and Newall, 2023). A scoping review of six peer-reviewed papers and two student theses found a likely relationship between gambling-related harm and cryptocurrency use (Johnson, Co, *et al*., 2023). Subsequently published work found a significant overlap between gambling-related harm and purchase of cryptoassets (Lopez-Gonzalez and Petrotta, 2024). A total of 40% of cryptoasset users in the UK self-identified that they purchased products as a gamble (Financial Conduct Authority, 2023b) and a qualitative analysis of Reddit messages on r/cryptocurrency found users were aware of the significant overlap between cryptocurrency trading and gambling, and discussed its impact on their mental health (Johnson, Stjepanović, *et al*., 2023). Although these studies are largely based in high-income countries, cryptocurrency ownership has the highest prevalence in low- and middle-income countries (Feyen *et al*., 2022). Cryptocurrencies also facilitate anonymous payments for illicit goods, such as weaponry and illicit drugs, undermining public health policies to reduce harm from these products (Foley *et al*., 2019).

## REVIEW OF CRYPTOCURRENCIES AGAINST COMMERCIAL DETERMINANTS OF HEALTH FRAMEWORK

Having summarized the more well-recognized harms of cryptocurrency, this research assesses cryptocurrency against the framework set out in the recent Lancet series on commercial determinants of health (Lacy-Nichols *et al*., 2023). This framework is particularly useful for cryptocurrencies, because it involves multi-national commercial actors, and the pathways by which these products may damage health are particularly complex. The focus is on cryptocurrencies as the predominant and best-researched form of cryptoasset. All headings and all relevant subheadings of the framework have been applied. This is intended to demonstrate the range of activities that cryptocurrency companies carry out and to assess whether, how and to what extent cryptocurrency actors could influence health outcomes.

### Practices

#### Political

There is significant evidence of cryptocurrency firms seeking to gain political influence through direct donations. Sam Bankman-Fried, one of the leaders of the FTX cryptocurrency exchange, was convicted of fraud, money laundering and campaign finance law violations in 2023 (Reuters, 2023). In 2022, Bankman-Fried was the seventh-largest registered individual political donor in the USA, donating more than $40 million (OpenSecrets, 2024). Donald Trump’s 2024 presidential campaign received $131 million in donations from cryptocurrency-backed organizations (CNN, 2024). Nearly all donations to the UK’s Reform UK party (formerly Brexit Party) from 2019 to 2023 were from cryptocurrency hedge fund manager Christoper Harborne, who also donated to the governing Conservative party (The Electoral Commission, 2024). Despite the risks cryptocurrencies pose to members of the public, some governments across the world have sought to position themselves as leaders of this new technology. El Salvador’s government adopted Bitcoin as legal tender, despite the hugely volatile nature of the currency posing a risk to everyday Salvadorians (The Economist, 2022), the UK Conservative government declared its intention to become a ‘global cryptoasset technology hub’ (HM Treasury, 2022) and Donald Trump pledged to create a ‘national cryptocurrency reserve’ (CNN, 2024).

#### Marketing

Cryptocurrency firms have invested extremely large sums of money in advertising in a very short space of time. Following the well-trodden path of tobacco, alcohol, HFSS and gambling companies (Ireland *et al*., 2019), in recent years cryptocurrency firms have taken on seismic contracts with sports organizations across the world. Tom Brady, the decorated American football player, was reportedly paid $55 million to promote cryptocurrency for 20 hours a year for three years (The New York Times, 2023). All 10 Formula One teams were sponsored by cryptocurrency companies in 2022 (Bloomberg, 2023). A total of 19 of 20 Premier League teams in 2022/2023 held a cryptocurrency or blockchain-related sponsor, and nearly all the sponsor product offerings plummeted in value soon after sponsorship deals began (D’Urso, 2022). Crypto-casino sponsors have had a strong presence in English football (Torrance *et al*., 2023) and cryptocurrencies have also partially filled the void of advertising left by gambling sponsors in Spanish football (Newall and Xiao, 2021). When sponsoring cryptocurrency corporations collapse, this can result in financial harm to the organizations that have taken on these sponsors (The Sponsor, 2023). It is not just the size of the advertising budget that has been notable, as many cryptocurrency firms have been found to have flaunted advertising standards rules. In the UK, the Advertising Standards Agency found that some firms trivialized investing in risky assets, employed ‘fear of missing out’ to induce investment, and falsely implied products were regulated (Financial Times (UK), 2021; ASA and CAP, 2022).

#### Supply chain and waste

One of the key activities of many cryptocurrencies is ‘mining’, harnessing huge computing power to create new digital coins (Financial Conduct Authority, 2024). This requires huge consumption of electricity; the US Energy Information Administration estimates that 0.6–2.3% of all US electricity demand in 2023 could be attributed to bitcoin mining—enough to power between three to six million homes (U.S. Energy Information Administration (EIA), 2023). This exacerbates the climate crisis that will disproportionately affect communities and nations already experiencing poorer health (Howson and de Vries, 2022).

#### Financial

Cryptocurrency firms have engaged in several questionable practices in raising capital. At the extreme end are ‘pump-and-dump’ schemes, where a small group of people with holdings in a specific cryptocurrency go out to recruit new investors with the plan of selling once the price has been hyped artificially high, leaving new investors severely out of pocket (Torrance *et al*., 2023). Other practices include sleight-of-hand, where firms appear to be primarily selling a non-cryptocurrency product (such as sport fan engagement tokens) but require purchases to be made in their cryptocurrency, which they market separately as an investment opportunity (Calladine, 2024). The lack of regulation of cryptocurrency has enabled new companies to start up and offer products without the significant oversight required of regular financial products (House of Commons Treasury Committee, 2023).

### Portfolio

Gambling has increasingly been established as a public health issue (van Schalkwyk *et al*., 2021). The similarities between gambling and unbacked cryptocurrency are numerous. They lack intrinsic value or clear social benefit, carry the potential for extreme financial gains or losses, and most consumers end up losing money (Davies and Ferris, 2022; Newall and Weiss-Cohen, 2022; House of Commons Treasury Committee, 2023). A series of crypto-casinos have been launched across the globe where the primary form of payment accepted is cryptocurrency, essentially creating a multi-layered gambling product; their websites frequently fail to provide even basic gambling harm protections (Andrade *et al*., 2022).

An evidence review of gambling found it causes health harm, both through the wider determinants of health and specific health harm such as deaths from suicide, depression, alcohol dependence and illicit drug use (Public Health England, 2021). Whereas these same harms cannot be directly inferred from gambling to cryptocurrency, a review of cryptocurrencies as gamblified financial products identified three gambling-related risks specific to cryptocurrency; market manipulation, behavioural similarities with gambling and social media-driven herd behaviour (Andrade and Newall, 2023). The language of protection against cryptocurrency ‘individualizes’ the harm caused by cryptocurrencies, such as exhorting consumers to make more ‘informed investment decisions’ (Financial Conduct Authority, 2023a), mirroring the ‘responsible gambling’ messages propagated by the gambling industry (Livingstone and Rintoul, 2020). Research on cryptocurrencies sponsored by the gambling industry has been published (Louderback *et al*., 2024); it is important to have non-conflicted study of the gamblified nature of cryptocurrency.

Cryptocurrency has provided opportunities for hundreds of small- and large-scale frauds, many of which affect those from low- and middle-income countries. For example, the Mirror Trading International fraud, based in South Africa, lost $1.7 billion of investor money, and the PlusToken scam took over $2 billion from victims largely based in China (Chainalysis, 2022). Financial losses and debt are linked to a host of poorer physical and mental health outcomes (Benzeval *et al*., 2014). Even with above-board ventures, smaller investors are the worst affected by market turmoil in cryptocurrency (Cornelli *et al*., 2023).

Cryptocurrency products have been around for as long as the iPhone (Financial Conduct Authority, 2024) but whereas the smartphone has seen rapid development into a ubiquitous global product, volatile cryptocurrencies remain largely a speculation vehicle with very little use as a currency (Nguyen and Watson, 2023; US Federal Reserve, 2023). This includes the purchase of illicit drugs and facilitation of illegal transactions, as cryptocurrencies offer greater anonymity than transactions in fiat (regular) currencies and thus make it more difficult for law enforcement agencies to track (Foley *et al*., 2019). This can undermine public health policies to reduce harms from these products. It is possible that the blockchain technology that cryptocurrencies are based on will eventually offer a faster and more decentralized form of payment that is not tied to speculation, or for contracting. However, the longer it takes for this potential to be realised (if it ever is), the longer the potential cumulative public health harms of cryptocurrency grow.

### Resources

#### Geographic range

There are several examples of cryptocurrency firms that base themselves in one country, seek sponsorship in another and concentrate their actions elsewhere. For example, Titan Capital Markets, under investigation for fraud, took up a financial license in Australia and sponsored English Premier League team Fulham FC, but spent its time soliciting investment in West Africa (Calladine, 2024). Companies may use the legitimacy associated with institutions and companies in high-income countries to run frauds in low- and middle-income countries. Because of patchy regulation and the international nature of cryptocurrency transactions, it is difficult for regulators to cope with companies that have strategically based themselves in third-party locations (Parliament of Australia, 2022).

### Organization

Because cryptocurrencies are based on principles of decentralization and anonymity, it can be very hard to ascertain who has power within any given structure (Myeong *et al*., 2023). For example, some cryptocurrencies operate through decentralized autonomous organizations (DAOs), which means that voting and financial decisions are handled through blockchain (Barbereau and Bodó, 2023). This has the secondary effect of making it very difficult to pursue legal recourse if a cryptocurrency overseen by a DAO is involved in illegal practices (Barbereau and Bodó, 2023). Other cryptocurrency organizations, including those that have taken on huge advertising contracts with top-tier sports clubs, withhold basic information on ownership, geographical location and organization structure, or even publish falsified information on their organizational leadership, making legal action for fraud victims very difficult to pursue (Calladine, 2024).

### Transparency

Transparency is a paradoxical concept in cryptocurrency. On the one hand, transactions on the blockchain should be distributed across every ledger holder, maximizing the transparency of transactions (Financial Conduct Authority, 2024). On the other, it is possible for those involved to largely shield their identities, not just from other users, but from law enforcement and state apparatus (Foley *et al*., 2019).

Cryptocurrencies have found themselves criticized repeatedly for mis-selling of products. It has been common for firms to try to demonstrate a corporate focus on ‘Web-3’ engagement products at a corporate or sponsorship level, whilst focusing on offering cryptocurrency for speculative purposes to members of the public (Calladine, 2024). It has been demonstrated that a great proportion of cryptocurrency users do not understand the product they are using; less than half of those who have owned cryptoassets in the UK self-reported that they had a good understanding of the underlying technology (Financial Conduct Authority, 2023b).

## DISCUSSION

### Summary of findings

The evidence for specific health-related harms of cryptocurrencies is nascent, given their relative novelty and complexity. Few longitudinal or medium- to long-term repeated cross-sectional studies exist that allow for causal inference, and those that exist focus on limited outcomes (Johnson, Co, *et al*., 2023). However, this assessment of cryptocurrencies as a commercial determinant of health highlights significant concerns about the health-harming practices of cryptocurrency firms, including providing extremely risky, gamblified products, considerable political lobbying, pervasive and untrue marketing strategies, and operations that contribute to substantial unnecessary energy consumption. The tactics employed by many firms, ranging from overpromising of financial rewards to cut-and-dried fraud, pose substantial risks to the public. The shared similarities between gambling and speculative investment in unbacked cryptocurrencies are a strong signifier that public health harms can arise from their volatility and speculative nature. Despite the promise of blockchain technology for decentralized payment systems, the lack of progress towards such applications over more than a decade suggests significantly increased regulation is required to manage the risks of ongoing public health costs associated with cryptocurrency speculation.

### Implications

It is not possible to predict what the next harmful gamblified product will be, but the example of cryptocurrency shows it will likely have many of the following features; very large-scale sponsorship deals with sports clubs, celebrity endorsements, gambling-like features, high-risk products with marketing aimed at youth, and a desire to operate outside of usual regulatory frameworks.

Public health researchers and professionals may be loath to criticize new technological developments like cryptocurrency for several reasons. Some may fear being labelled a Luddite, a blocker of exciting technological progress. Some may worry this is ‘straying out of lane’ from public health into finance and economics. This analysis is on one particular new technology, cryptocurrencies. If new technologies or products are using the corporate ‘playbook’ of health-harming industries (Lacy-Nichols *et al*., 2022) and display similarities to pre-existing harmful products, then it is incumbent on public health researchers with experience of commercial determinants of health to speak loudly and to draw parallels with other industries who have used similar methods to draw in the public, and to urge regulation in marketing and availability, even if long-term health harm cannot yet be demonstrated. If a technology does develop to a point to which its benefits are shown to outweigh its costs, regulations can always be amended.

The extensive promotion, proliferation and scale of cryptocurrency use make this topic an urgent research priority. There is a well-developed evidence base using qualitative methods that have reported the effects of gambling advertising, the harm to gamblers and the harm to family members (McCarthy *et al*., 2020; Schalkwyk *et al*., 2021; Marko *et al*., 2023; Thomas *et al*., 2023); this could be replicated for cryptocurrency. Furthermore, as cryptocurrency investment has been demonstrated to have such similarity to gambling (Andrade and Newall, 2023) future studies on gambling should consider including cryptocurrency use in their outcomes of interest. Studies in low- and middle-income countries are of particular importance, given the higher prevalence of cryptocurrency ownership in these settings (Feyen *et al*., 2022). Studying the epidemiological links between cryptocurrency and illicit drugs, weaponry and other illicit harmful products would be informative. It is of vital importance that studies are conducted by authors who are free of conflicts of interest from both cryptoasset industries and gambling industries (Lacy-Nichols *et al*., 2022). Given that population-level regulation has the greatest efficacy in reducing harm from commercial activity, a high priority should be placed on studying the effectiveness of current national and international regulation, and ways this can be strengthened.

## CONCLUSIONS

When analysed through the commercial determinants of health framework, cryptocurrency is shown to be a potent threat to global population health. Public health researchers and practitioners with experience in the commercial determinants of health must continue to research and advocate for protections against cryptocurrencies. Looking ahead, regulators and governments across the globe must prepare legislation that protects against future threats that straddle the worlds of gambling and finance.

## Data Availability

The data underlying this article are available in the article.
